# 2,4-Bis(3-fluoro­phen­yl)-3-aza­bicyclo­[3.3.1]nonan-9-one

**DOI:** 10.1107/S1600536808024690

**Published:** 2008-08-06

**Authors:** P. Parthiban, K. Thirumurugan, V. Ramkumar, S. Pazhamalai, Yeon Tae Jeong

**Affiliations:** a Division of Image Science and Information Engineering, Pukyong National University, Busan 608 739, Republic of Korea; b Orchid Chemical and Pharmaceuticals Ltd, R&D Centre, Sholinganallur, Chennai, Tamilnadu, India; c Department of Chemistry, IIT Madras, Chennai, TamilNadu, India; d Department of Chemistry, Annamalai University, Annamalai Nagar, Chidambaram, Tamilnadu, India

## Abstract

The title compound, C_20_H_19_F_2_NO, exhibits a chair–chair conformation, with the aryl groups in the heterocycle in equatorial orientations and oriented at an angle of 33.35 (3)° to one another. A crystallographic mirror plane, passing through the N atom, the C and O atoms of the carbonyl group and the C atom in the 7-position, bis­ects the mol­ecule. The mol­ecular structure is stabilized by one C—H⋯N inter­action and the crystal structure is stabilized by a weak C—H⋯π inter­action.

## Related literature

For related literature, see: Barker *et al.* (2005 ▶); Dunitz *et al.* (1997 ▶); Evans *et al.* (1997 ▶); Jeyaraman *et al.* (1981 ▶); Padegimas *et al.* (1972 ▶); Smith-Verdier *et al.* (1983 ▶); Web *et al.* (1967 ▶); Wiechert *et al.* (1997 ▶); Cremer & Pople (1975 ▶); Ramachandran *et al.* (2007 ▶); Vijayalakshmi *et al.* (2000 ▶).
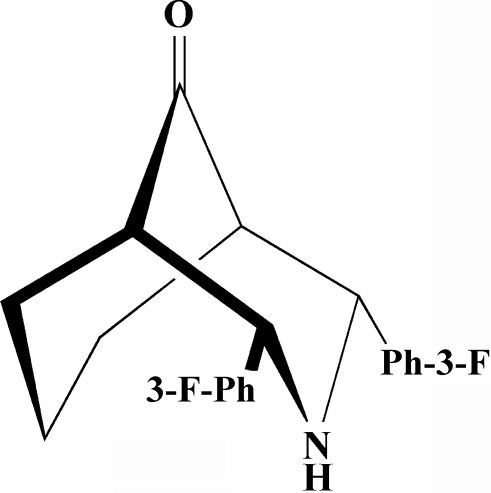


## Experimental

### 

#### Crystal data

C_20_H_19_F_2_NO
*M* *_r_* = 327.36
Orthorhombic, 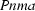

*a* = 7.3844 (2) Å
*b* = 21.5172 (10) Å
*c* = 10.2608 (4) Å
*V* = 1630.36 (11) Å^3^
*Z* = 4
Mo *K*α radiation
μ = 0.10 mm^−1^
*T* = 298 (2) K
0.35 × 0.19 × 0.15 mm

#### Data collection

Bruker APEXII CCD area-detector diffractometer
Absorption correction: multi-scan (*SADABS*; Bruker, 1999 ▶) *T*_min_ = 0.967, *T*_max_ = 0.986
11640 measured reflections
2069 independent reflections
1507 reflections with *I* > 2σ(*I*)
*R*_int_ = 0.023

#### Refinement

*R*[*F* ^2^ > 2σ(*F* ^2^)] = 0.051
*wR*(*F* ^2^) = 0.154
*S* = 1.03
2069 reflections
118 parameters
H atoms treated by a mixture of independent and constrained refinement
Δρ_max_ = 0.27 e Å^−3^
Δρ_min_ = −0.26 e Å^−3^

### 

Data collection: *APEX2* (Bruker–Nonius, 2004 ▶); cell refinement: *APEX2*; data reduction: *SAINT-Plus* (Bruker–Nonius, 2004 ▶); program(s) used to solve structure: *SHELXS97* (Sheldrick, 2008 ▶); program(s) used to refine structure: *SHELXL97* (Sheldrick, 2008 ▶); molecular graphics: *ORTEP-3* (Farrugia, 1997 ▶); software used to prepare material for publication: *SHELXL97*.

## Supplementary Material

Crystal structure: contains datablocks global, I. DOI: 10.1107/S1600536808024690/bx2166sup1.cif

Structure factors: contains datablocks I. DOI: 10.1107/S1600536808024690/bx2166Isup2.hkl

Additional supplementary materials:  crystallographic information; 3D view; checkCIF report

## Figures and Tables

**Table 1:** Hydrogen-bond geometry (Å, °)

*D*—H⋯*A*	*D*—H	H⋯*A*	*D*⋯*A*	*D*—H⋯*A*
C4—H4*B*⋯N1	0.97	2.48	2.923 (3)	108
C11—H11⋯*Cg*1^i^	0.93	2.93	3.862 (2)	175

Symmetry code: (i) 
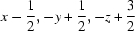
. Cg1 is the centroid of the C6–C11 ring.

**Fig. 1.**
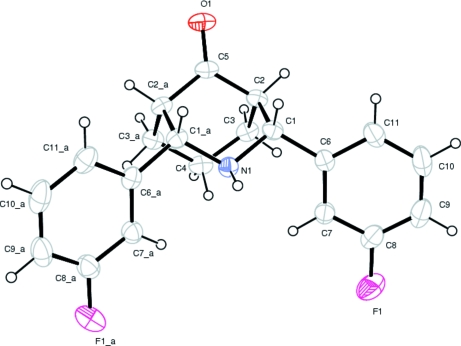
ORTEP of the molecule with atoms represented as 30% probability ellipsoids.[symmetry code: _a: x,1/2-y, z]

## supplementary crystallographic information

### Comment

Azabicyclic ketones are important class of heterocycles due to their broad spectrum biological activities (Jeyaraman & Avila, 1981; Barker *et al.*, 2005). Moreover, the fluorine substituted organic molecules are valuable due to the significance of C—F bonds in some bioorganic systems (Evans & Seddon, 1997; Dunitz & Tailor, 1997). Hence, the biological significance of the hydrogen bonds involving fluorine atom have attracted much attention (Ramachandran *et al.*, 2007;Wiechert *et al.*, 1997). Owing to the diverse possibilities in conformations, *viz*.,chair-chair (Vijayalakshmi *et al.*, 2000), chair-boat (Smith-Verdier *et al.*, 1983) and boat-boat (Padegimas & Kovacic, 1972) for the azabicycle, the present crystal study was undertaken to explore the conformation, stereochemistry and bondings in the title compound.The study of torsion angles, asymmetry parameters and least-squares plane calculation of the title compound shows that the piperidine ring adopts near ideal chair conformation with the deviation of ring atoms N1 and C5 from the C1/C2/C1a/C2a plane by 0.659 (3) and -0.693 (3)Å respectively, the q(2) and q(3) are 0.0165 (16) and -0.6032 (16) Å. The total puckering amplitude, Q_T_ = 0.6034 (16)Å and θ = 178.44 (15)°. The cyclohexane ring deviate from the ideal chair conformation by the deviation of ring atoms C4 and C5 from the C2/C3/C2a/C3a plane by 0.527 (4) and - 0.727 (3)Å respectively. For the cyclohexane part, the q(2) and q(3) are 0.1472 (18) and -0.5470 (17)Å respectively. The total puckering amplitude, Q_T_ = 0.5664 (17) and θ =164.95 (18)° (Cremer & Pople, 1975; Web & Becker, 1967).Hence, the title compound C_20_ H_19_F_2_NO, exists in twin-chair conformation with equatorial orientations of the 3-fluorophenyl groups on the heterocycle and are orientated at an angle of 33.35 (3) ° to each other. The torsion angles of C5—C2—C1—C6 and its mirror plane C5—C2a—C1a—C6a is 178.51 (6)°.

### Experimental

In a warm solution of ammonium acetate (0.075 mol) in 50 ml of absolute ethanol, a mixture of cyclohexanone (0.05 mol) and *meta* fluorobenzaldehyde (0.1 mol) was added and very gently warmed on a hot plate till the yellow color formed during the mixing of the reactants and stirred to the formation of the product. Then, the compound was separated by filtration and washed with 1:5 ethanol-ether mixture. Thus, the separated crude compound was purified by recrystallization from ethanol to obtain the colorless diffraction quality crystals of 2,4-bis (3-fluorophenyl)-3-azabicyclo [3.3.1]nonan-9-one.

### Refinement

Nitrogen H atoms were located in a difference Fourier map and refined isotropically. Other hydrogen atoms were fixed geometrically and allowed to ride on the parent carbon atoms,with aromatic C—H =0.93 Å, aliphatic C—H = 0.98Å and methylen C—H = 0.97 Å. The displacement parameters were set for phenyl,methylen and aliphatic H atoms at *U*_iso_(H) = 1.2*U*_eq_(C).

### Crystal data

**Table:**

C_20_H_19_F_2_NO	*F*_000_ = 688
*M**_r_* = 327.36	*D*_x_ = 1.334 Mg m^−^^3^
Orthorhombic, *P**n**m**a*	Mo *K*α radiation λ = 0.71073 Å
Hall symbol: -P 2ac 2n	Cell parameters from 3860 reflections
*a* = 7.3844 (2) Å	θ = 2.2–27.8º
*b* = 21.5172 (10) Å	µ = 0.10 mm^−^^1^
*c* = 10.2608 (4) Å	*T* = 298 (2) K
*V* = 1630.36 (11) Å^3^	Needle, colourless
*Z* = 4	0.35 × 0.19 × 0.15 mm

### Data collection

**Table:**

Bruker APEXII CCD area-detector diffractometer	2069 independent reflections
Radiation source: fine-focus sealed tube	1507 reflections with *I* > 2σ(*I*)
Monochromator: graphite	*R*_int_ = 0.023
*T* = 298(2) K	θ_max_ = 28.3º
φ and ω scans	θ_min_ = 1.9º
Absorption correction: multi-scan(SADABS; Bruker, 1999)	*h* = −9→9
*T*_min_ = 0.967, *T*_max_ = 0.986	*k* = −28→27
11640 measured reflections	*l* = −13→13

### Refinement

**Table:**

Refinement on *F*^2^	Secondary atom site location: difference Fourier map
Least-squares matrix: full	Hydrogen site location: inferred from neighbouring sites
*R*[*F*^2^ > 2σ(*F*^2^)] = 0.051	H atoms treated by a mixture of independent and constrained refinement
*wR*(*F*^2^) = 0.154	*w* = 1/[σ^2^(*F*_o_^2^) + (0.0736*P*)^2^ + 0.4778*P*] where *P* = (*F*_o_^2^ + 2*F*_c_^2^)/3
*S* = 1.03	(Δ/σ)_max_ < 0.001
2069 reflections	Δρ_max_ = 0.27 e Å^−^^3^
118 parameters	Δρ_min_ = −0.25 e Å^−^^3^
Primary atom site location: structure-invariant direct methods	Extinction correction: none

### Special details

**Table:**

Geometry. All e.s.d.'s (except the e.s.d. in the dihedral angle between two l.s. planes)are estimated using the full covariance matrix. The cell e.s.d.'s are takeninto account individually in the estimation of e.s.d.'s in distances, anglesand torsion angles; correlations between e.s.d.'s in cell parameters are onlyused when they are defined by crystal symmetry. An approximate (isotropic)treatment of cell e.s.d.'s is used for estimating e.s.d.'s involving l.s. planes.
Refinement. Refinement of *F*^2^ against ALL reflections. The weighted *R*-factor *wR* andgoodness of fit *S* are based on *F*^2^, conventional *R*-factors *R* are basedon *F*, with *F* set to zero for negative *F*^2^. The threshold expression of*F*^2^ > σ(*F*^2^) is used only for calculating *R*-factors(gt) *etc*. and isnot relevant to the choice of reflections for refinement. *R*-factors basedon *F*^2^ are statistically about twice as large as those based on *F*, and *R*-factors based on ALL data will be even larger.

### Fractional atomic coordinates and isotropic or equivalent isotropic displacement parameters (Å^2^)

**Table:**

	*x*	*y*	*z*	*U*_iso_*/*U*_eq_	
C1	0.9282 (2)	0.30645 (7)	0.60735 (13)	0.0415 (4)	
H1	0.8910	0.3052	0.6990	0.050*	
C2	0.75387 (19)	0.30789 (7)	0.52202 (15)	0.0444 (4)	
H2	0.6813	0.3441	0.5468	0.053*	
C3	0.7856 (2)	0.30939 (8)	0.37344 (15)	0.0482 (4)	
H3A	0.6707	0.3166	0.3302	0.058*	
H3B	0.8646	0.3441	0.3529	0.058*	
C4	0.8693 (3)	0.2500	0.3196 (2)	0.0501 (6)	
H4A	0.8565	0.2500	0.2255	0.060*	
H4B	0.9978	0.2500	0.3394	0.060*	
C5	0.6479 (3)	0.2500	0.5518 (2)	0.0460 (5)	
C6	1.0432 (2)	0.36362 (7)	0.58774 (14)	0.0436 (4)	
C7	1.1855 (2)	0.36471 (8)	0.49973 (17)	0.0509 (4)	
H7	1.2153	0.3294	0.4522	0.061*	
C8	1.2815 (2)	0.41854 (9)	0.4838 (2)	0.0603 (5)	
C9	1.2457 (3)	0.47171 (9)	0.5505 (2)	0.0723 (6)	
H9	1.3139	0.5075	0.5371	0.087*	
C10	1.1056 (3)	0.47077 (9)	0.6385 (2)	0.0767 (7)	
H10	1.0775	0.5065	0.6853	0.092*	
C11	1.0059 (3)	0.41717 (8)	0.65820 (19)	0.0599 (5)	
H11	0.9128	0.4170	0.7193	0.072*	
F1	1.42123 (18)	0.41845 (6)	0.39681 (15)	0.0962 (5)	
N1	1.0305 (2)	0.2500	0.57975 (17)	0.0395 (4)	
O1	0.4936 (2)	0.2500	0.59191 (18)	0.0638 (5)	
H1A	1.127 (3)	0.2500	0.629 (2)	0.047 (6)*	

### Atomic displacement parameters (Å^2^)

**Table:**

	*U*^11^	*U*^22^	*U*^33^	*U*^12^	*U*^13^	*U*^23^
C1	0.0426 (8)	0.0480 (9)	0.0338 (7)	0.0033 (6)	0.0003 (6)	−0.0020 (6)
C2	0.0364 (7)	0.0515 (10)	0.0454 (8)	0.0071 (6)	0.0012 (6)	−0.0016 (7)
C3	0.0432 (8)	0.0592 (10)	0.0423 (8)	−0.0006 (7)	−0.0067 (6)	0.0080 (7)
C4	0.0439 (11)	0.0734 (16)	0.0331 (10)	0.000	−0.0014 (9)	0.000
C5	0.0361 (11)	0.0656 (15)	0.0362 (10)	0.000	0.0020 (8)	0.000
C6	0.0450 (8)	0.0426 (9)	0.0431 (7)	0.0051 (6)	−0.0104 (6)	−0.0028 (6)
C7	0.0511 (9)	0.0455 (9)	0.0560 (9)	−0.0018 (7)	−0.0026 (7)	−0.0016 (7)
C8	0.0528 (10)	0.0552 (11)	0.0731 (12)	−0.0072 (8)	−0.0096 (9)	0.0110 (9)
C9	0.0659 (12)	0.0455 (11)	0.1056 (17)	−0.0076 (9)	−0.0312 (12)	0.0070 (11)
C10	0.0815 (14)	0.0464 (11)	0.1024 (16)	0.0107 (10)	−0.0309 (13)	−0.0217 (11)
C11	0.0610 (10)	0.0555 (11)	0.0632 (10)	0.0109 (8)	−0.0095 (9)	−0.0158 (8)
F1	0.0816 (9)	0.0895 (10)	0.1176 (11)	−0.0267 (7)	0.0191 (8)	0.0147 (8)
N1	0.0367 (9)	0.0412 (10)	0.0407 (9)	0.000	−0.0063 (7)	0.000
O1	0.0408 (9)	0.0867 (14)	0.0639 (11)	0.000	0.0146 (8)	0.000

### Geometric parameters (Å, °)

**Table:**

C1—N1	1.4585 (17)	C5—C2^i^	1.5025 (19)
C1—C6	1.508 (2)	C6—C7	1.386 (2)
C1—C2	1.557 (2)	C6—C11	1.388 (2)
C1—H1	0.9800	C7—C8	1.368 (2)
C2—C5	1.5025 (19)	C7—H7	0.9300
C2—C3	1.543 (2)	C8—C9	1.359 (3)
C2—H2	0.9800	C8—F1	1.364 (2)
C3—C4	1.523 (2)	C9—C10	1.373 (3)
C3—H3A	0.9700	C9—H9	0.9300
C3—H3B	0.9700	C10—C11	1.383 (3)
C4—C3^i^	1.523 (2)	C10—H10	0.9300
C4—H4A	0.9700	C11—H11	0.9300
C4—H4B	0.9700	N1—C1^i^	1.4585 (17)
C5—O1	1.212 (2)	N1—H1A	0.87 (2)
			
N1—C1—C6	111.20 (12)	O1—C5—C2	123.98 (8)
N1—C1—C2	109.63 (12)	O1—C5—C2^i^	123.98 (8)
C6—C1—C2	111.97 (12)	C2—C5—C2^i^	112.01 (17)
N1—C1—H1	108.0	C7—C6—C11	118.42 (16)
C6—C1—H1	108.0	C7—C6—C1	121.85 (13)
C2—C1—H1	108.0	C11—C6—C1	119.73 (15)
C5—C2—C3	107.30 (14)	C8—C7—C6	119.02 (16)
C5—C2—C1	107.44 (13)	C8—C7—H7	120.5
C3—C2—C1	115.49 (12)	C6—C7—H7	120.5
C5—C2—H2	108.8	C9—C8—F1	118.52 (17)
C3—C2—H2	108.8	C9—C8—C7	123.5 (2)
C1—C2—H2	108.8	F1—C8—C7	117.96 (17)
C4—C3—C2	113.73 (14)	C8—C9—C10	117.71 (19)
C4—C3—H3A	108.8	C8—C9—H9	121.1
C2—C3—H3A	108.8	C10—C9—H9	121.1
C4—C3—H3B	108.8	C9—C10—C11	120.63 (18)
C2—C3—H3B	108.8	C9—C10—H10	119.7
H3A—C3—H3B	107.7	C11—C10—H10	119.7
C3^i^—C4—C3	114.07 (18)	C10—C11—C6	120.68 (19)
C3^i^—C4—H4A	108.7	C10—C11—H11	119.7
C3—C4—H4A	108.7	C6—C11—H11	119.7
C3^i^—C4—H4B	108.7	C1^i^—N1—C1	112.78 (16)
C3—C4—H4B	108.7	C1^i^—N1—H1A	108.1 (8)
H4A—C4—H4B	107.6	C1—N1—H1A	108.1 (8)
			
N1—C1—C2—C5	57.58 (16)	C2—C1—C6—C11	84.78 (17)
C6—C1—C2—C5	−178.52 (13)	C11—C6—C7—C8	−1.2 (2)
N1—C1—C2—C3	−62.09 (17)	C1—C6—C7—C8	178.18 (15)
C6—C1—C2—C3	61.81 (17)	C6—C7—C8—C9	0.4 (3)
C5—C2—C3—C4	−52.65 (18)	C6—C7—C8—F1	179.86 (15)
C1—C2—C3—C4	67.10 (18)	F1—C8—C9—C10	−179.43 (17)
C2—C3—C4—C3^i^	44.0 (2)	C7—C8—C9—C10	0.1 (3)
C3—C2—C5—O1	−113.3 (2)	C8—C9—C10—C11	0.4 (3)
C1—C2—C5—O1	122.0 (2)	C9—C10—C11—C6	−1.2 (3)
C3—C2—C5—C2^i^	65.0 (2)	C7—C6—C11—C10	1.6 (3)
C1—C2—C5—C2^i^	−59.8 (2)	C1—C6—C11—C10	−177.76 (16)
N1—C1—C6—C7	28.45 (19)	C6—C1—N1—C1^i^	175.53 (10)
C2—C1—C6—C7	−94.57 (16)	C2—C1—N1—C1^i^	−60.11 (18)
N1—C1—C6—C11	−152.20 (15)		

Symmetry codes: (i) *x*, −*y*+1/2, *z*.

### Hydrogen-bond geometry (Å, °)

**Table:**

*D*—H···*A*	*D*—H	H···*A*	*D*···*A*	*D*—H···*A*
C4—H4B···N1	0.97	2.48	2.923 (3)	108
C11—H11···Cg1^ii^	0.93	2.93	3.862 (2)	175

Symmetry codes: (ii) *x*−1/2, −*y*+1/2, −*z*+3/2.
